# Nitro-Glycerine in Epilepsy

**Published:** 1877-01

**Authors:** 


Nitro-Glycerine in Epilepsy.—Dr. A. McLane Hamilton, in an excellent article upon the therapeutics of epilepsy, (*Chicago Med. Jour. and Examiner)*, states that he has found tri-nitro-glycerine as powerful a medicinal agent as it is an explosive. The tenth part of a drop touched to the tongue is sufficient in a space of time which is almost inappreciable to produce a rapid cerebral hyperæmia. The face is flushed, the eyes become bright, and the temporal vessels throb, while at the same time there are the marked sensations of fullness. It produces more lasting congestions than does amyl nitrite, is much safer, and he has found it to act much better as an abortant of the epileptic seizure than the latter. Any good pharmacist can prepare a solution containing one drop to ten of alcohol. This can be further diluted so that ten drops of alcohol shall contain one-tenth of a drop of the nitro-glycerine. It may be kept safely in this way, for alcohol prevents its explosion. A dose of a tenth of a drop is sufficient in the majority of cases.

